# From digital traces to public vaccination behaviors: leveraging large language models for big data classification

**DOI:** 10.3389/frai.2025.1602984

**Published:** 2025-07-23

**Authors:** Yoo Jung Oh, Muhammad Ehab Rasul, Emily McKinley, Christopher Calabrese

**Affiliations:** ^1^Department of Communication, Michigan State University, East Lansing, MI, United States; ^2^Department of Communication, University of California, Davis, Davis, CA, United States; ^3^Department of Communication, Clemson University, Clemson, SC, United States

**Keywords:** artificial intelligence, large language models, LLMS, social media, COVID-19, vaccination

## Abstract

**Introduction:**

The current study leverages large language models (LLMs) to capture health behaviors expressed in social media posts, focusing on COVID-19 vaccine-related content from 2020 to 2021.

**Methods:**

To examine the capabilities of prompt engineering and fine-tuning approaches with LLMs, this study examines the performance of three state-of-the-art LLMs: GPT-4o, GPT-4o-mini, and GPT-4o-mini with fine-tuning, focusing on their ability to classify individuals’ vaccination behavior, intention to vaccinate, and information sharing. We then cross-validate these classifications with nationwide vaccination statistics to assess alignment with observed trends.

**Results:**

GPT-4o-mini with fine-tuning outperformed both GPT-4o and the standard GPT-4o-mini in terms of accuracy, precision, recall, and F1 score. Using GPT-4o-mini with fine-tuning for classification, about 9.84% of the posts (*N* = 36,912) included personal behavior related to getting the COVID-19 vaccine while a majority of posts (71.45%; *N* = 267,930) included information sharing about the virus. Lastly, we found a strong correlation (*r* = 0.76, *p* < 0.01) between vaccination behaviors expressed on social media and the actual vaccine uptake over time.

**Discussion:**

This study suggests that LLMs can serve as powerful tools for estimating real-world behaviors. Methodological and practical implications of utilizing LLMs in human behavior research are further discussed.

## 1 Introduction

The growing significance of digital communication has created new opportunities to analyze and understand health behaviors as they manifest online. In particular, recent advancements in artificial intelligence (AI) and large language models (LLMs) offer significant potential for extending our understanding of how people discuss and engage with health-related issues in these digital spaces. LLMs such as GPT-4 and GPT-4o (OpenAI et al., 2023; OpenAI, 2024) offer new methodological perspectives by providing sophisticated language analysis capabilities that can facilitate the development of more dynamic and scalable models for capturing health behaviors. For example, a recent study employed a pre-trained BERT model to assess stance toward vaccination among online comments, revealing the impact of persuasive health messaging on users’ attitudes toward vaccines on social media (Ji et al., 2024).

As LLMs become increasingly valuable in health communication research, several areas merit further exploration. Two common methods for adapting LLMs to specific tasks are prompt engineering and fine-tuning. Prompt engineering involves manipulating the prompt by including examples and instructions for the task at inference time, whereas fine-tuning involves updating the weights of a pre-trained model using a supervised dataset tailored to the specific task (White et al., 2023). While the individual strengths of prompt engineering and fine-tuning have been examined in prior work (e.g., Hu et al., 2024), hybrid approaches that combine these methods have received comparatively less attention. Further exploration is needed to evaluate the comparative performance and practical advantages of such hybrid strategies relative to other modeling approaches. In addition, although studies focused on classifying health attitudes and behaviors expressed in social media (e.g., Ji et al., 2024), relatively few assess the extent to which these classifications correspond with real-world behaviors such as national vaccination uptake. Addressing these gaps will help advance both the methodological rigor and applied utility of LLM-based approaches in the study of online health communication.

To address the identified gaps, the current study focuses on capturing health behaviors by examining COVID-19 vaccine-related content shared in social media posts between 2020 and 2021, during the height of the pandemic. The study has the following aims. First, it examines the performance classifying health behaviors as expressed in social media data. Second, it evaluates how accurately these classifications mirror real-world vaccination behaviors by comparing the observed online trends with nationwide vaccination statistics. Lastly, the study outlines methodological and practical implications of integrating LLMs into health behavior research, highlighting their potential to enhance both the scope and the precision of investigations into online health behaviors.

## 2 Literature review

### 2.1 LLM-enhanced approaches to understand individuals’ health behaviors

Observing health behaviors at scale poses practical challenges, leading researchers to rely on self-reported measures such as intention, which may not always align with real-world behaviors. For example, a meta-analysis revealed that intentions had only a small-to-medium influence on subsequent actions, highlighting other important factors such as social reactions (Webb and Sheeran, 2006).

However, LLMs can effectively address these challenges by capturing individuals’ health behaviors in real-time at scale, thereby enhancing our understanding of their dynamic nature. Previous studies have employed LLMs to complement existing approaches. For instance, one study used early LLMs and crowdsourcing to classify the prevalence and temporal dynamics of descriptive norms surrounding tobacco and e-cigarette use in YouTube videos and tweets (Liu et al., 2019). This demonstrated the importance of examining norms over time, moving away from the individual level, for understanding their potential impact on online audiences. Similarly, BERT models have been used to classify key behavioral determinants related to COVID-19 vaccination within tweets, including positive and negative attitudes and behavioral intentions (Liu et al., 2021).

Since deep learning algorithms are trained on vast amounts of data, LLMs have the technical capacities to understand and generate human language across multiple modalities such as text, images, and audio (Tamkin et al., 2021) Recent work has argued LLMs’ reasoning and logical capabilities are similar to humans and can be employed to understand and identify complex human behaviors online. For instance, GPT models can generate persuasive political messages similar to humans (Bai et al., 2025), produce counter-arguments that reduce conspiracy beliefs (Costello et al., 2024), and respond to medical patients effectively (Ayers et al., 2023).

Moreover, the effectiveness of LLMs in text classification and understanding complex human behaviors demonstrates its ability to combat significant issues faced by society. For example, GPT models can be used to augment misinformation labeling capabilities due to their ability to successfully label true and false claims (Hu et al., 2024). Likewise, LLMs can successfully determine the credibility of social media posts by evaluating them for verified claims, matching human performance (Choi and Ferrara, 2024). These capabilities can support fact-checking organizations by enabling the systematic identification of both false and true claims.

A key methodological advantage of using LLMs is their ability to greatly reduce the time and effort needed for coding and annotation tasks while generating outputs comparable to human coders. LLMs show impressive performance when it comes to classification, with the most advanced model on par with human coders (Heseltine and Clemm von Hohenberg, 2024). For instance, one study found that the zero-shot accuracy of ChatGPT outperformed crowd workers for text-annotation tasks across multiple datasets (Gilardi et al., 2023). In some instances, LLMs such as GPT models even outperform human coders (Gilardi et al., 2023; Törnberg, 2023). Additionally, LLMs allow for increased reproducibility, particularly with tasks such as human annotations, although some challenges remain (Zhu et al., 2023).

### 2.2 Prompt engineering and fine-tuning

Further, prompt engineering and fine-tuning are crucial for optimizing LLM performance and tailoring models to specific research needs. Prompt engineering involves manipulating the prompt with examples and instructions of the task at inference time, whereas fine-tuning involves updating the weights of the pre-trained model by inputting a supervised dataset to the specific task (White et al., 2023). Existing research has found that prompt engineering can optimize LLM performance and provide stable outputs, which significantly broadens the scope of LLMs in different fields and contexts (Wang et al., 2024). In particular, two forms of prompting have been used by researchers: chain of thought (COT) prompting and tree of thoughts prompting (TOT). COT prompting is characterized by a series of reasoning steps as an input to the LLM, which results in improved outputs, particularly in complex scenarios such as math (Wei et al., 2022). In contrast, TOT prompting explores multiple paths of a problem, accounting for multiple thought processes to reach an outcome (Yao et al., 2023).

In addition to prompt engineering, fine-tuning is another approach utilized to improve the performance of LLMs. Fine-tuning focuses on training LLMs on specific data that is related to the task at hand. As such, in certain tasks, fine-tuned models can outperform LLMs that are trained via prompt engineering or few-shot learning (Bucher and Martini, 2024). Recent work has found that fine-tuned models outperformed LLMs trained using zero-shot/few-shot learning and COT prompting (Hu et al., 2024). However, there is no consensus among scholars about whether fine-tuned models should be preferred over prompted LLMs. One reason is because fine-tuned models are limited in scope as they are trained on specific data such as Wikipedia (Devlin et al., 2018), and are unable to handle requests that are beyond the data that they are trained on (Sheng et al., 2021). Instead, recent work suggests that fine-tuned and prompt approaches can complement each other, with hybrid approaches showing significant potential (Hu et al., 2024). For example, combining fine-tuned LLMs with few-shot learning or leveraging few-shot learning with adaptive fine-tuning has been shown to significantly improve the precision of the models (Chung et al., 2022). In this study, we explore the potential advantages between prompt engineering and fine-tuned modeling through comparing different LLMs for classifying vaccination behaviors on social media posts.

## 3 Methods

### 3.1 Data collection and procedures

**Figure 1:**
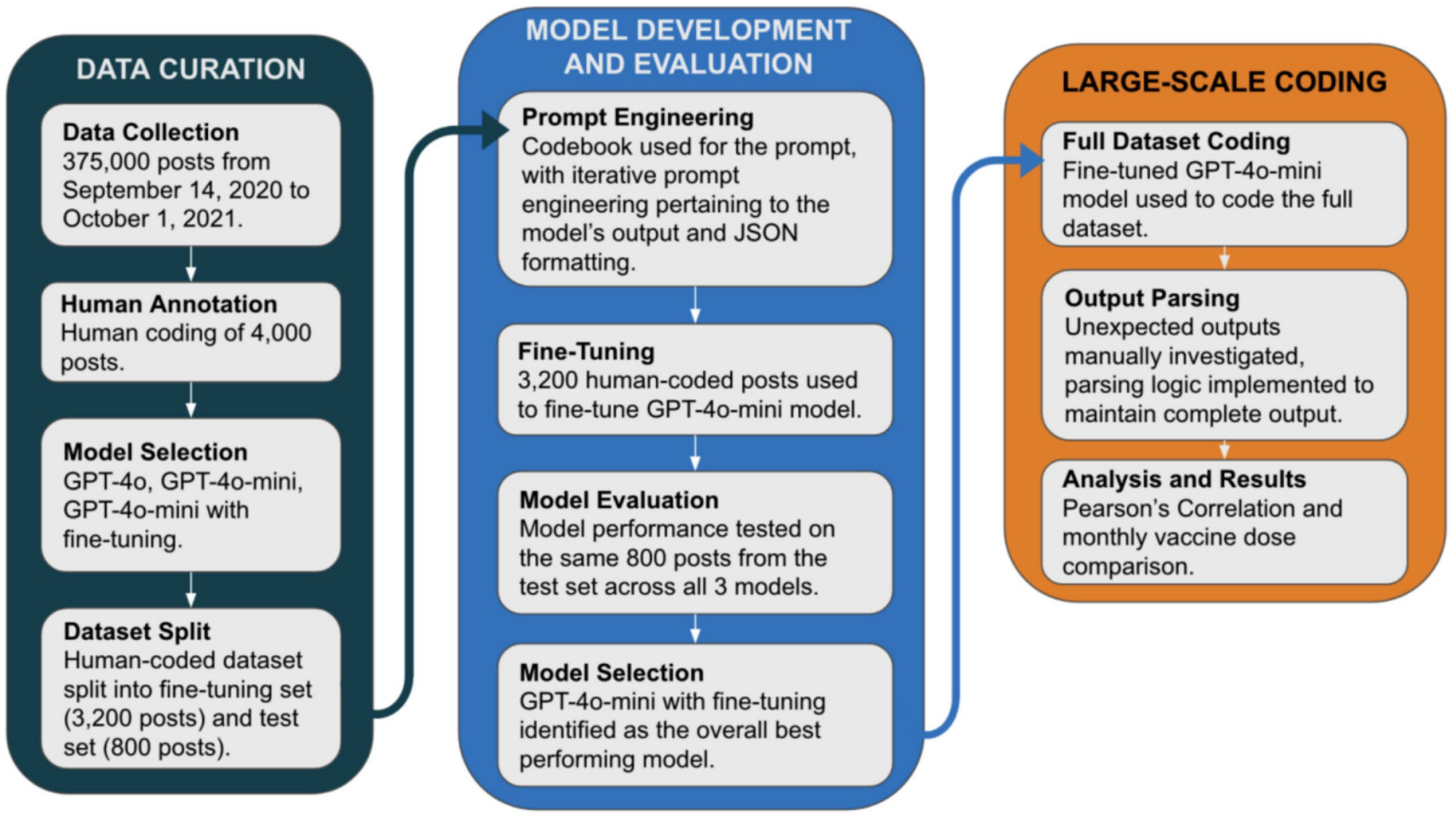
Method pipeline involving data curation, model development and evaluation, and large-scale coding procedures.

We utilized Twitter (now known as X) API to scrape tweets in English containing keywords related to the COVID-19 vaccine from September 14, 2020 to October 1, 2021. The keywords were selected to capture a comprehensive range of posts about COVID-19 vaccination, including different vaccine brands (e.g., Moderna, Pfizer, Johnson), variation of the term “vaccine” (e.g., vaccine, vaccines, vax), and variation of the word “vaccinate” (e.g., vaccinate, vaccinated, vaccinating). A systematic random sample of 7,000 tweets per week was selected to obtain a representative weekly sample of tweets; the final sample contained 375,000 tweets. We collected data starting 3 months before the first COVID-19 vaccine was administered in the US (December 14, 2020) and continued for 1 year to assess changes in public attitudes and behaviors related to health and vaccination. Figure 1 includes key steps for data curation, model development and evaluation, and large-scale coding.

The training dataset for coding personal behavior, intent, and information sharing composed of 4,000 tweets (i.e., 1% of the full dataset). Personal behavior was defined as an individual discussing their own vaccination experience or decision to get vaccinated against COVID-19. Intent was coded as positive, negative, or none, reflecting whether an individual expressed a (lack of) intention or likelihood to receive a COVID-19 vaccine in the future. Information sharing was categorized based on instances of an individual disseminating information about COVID-19 vaccinations. The coding scheme is presented in Supplementary Table 1. To assess reliability, a subset of 800 tweets was double-coded by both annotators. Inter-rater reliability was assessed using Krippendorff’s alpha and percent agreement, and the results were acceptable across all three categories: personal behavior (*α* = 0.767, 95.9%), intent (α = 0.710, 98.1%), and information sharing (α = 0.734, 89.5%). After establishing reliability, the remaining dataset was split evenly between coders, with any disagreements resolved through discussion.

### 3.2 Model selection and fine-tuning

Three OpenAI models were selected for evaluation in the coding task. These models were chosen due to their enhanced capabilities compared to earlier models (Liu et al., 2024), and their availability as of September 2024: GPT-4o, GPT-4o-mini, and GPT-4o-mini with fine-tuning (OpenAI, 2024). GPT-4o is a larger model, while GPT-4o-mini is a smaller, more computationally efficient version. All three models were accessed via custom Python scripts using API keys, and each was provided with the same prompt. This prompt included a revised version of the codebook used by human coders, along with strict output formatting instructions (see Supplementary Tables 1, 2). We employed a few-shot learning approach within the prompt by incorporating representative examples from the codebook for each category, ensuring coverage of both typical and edge cases in the data. The GPT-4o-mini model was fine-tuned using 3,200 of the 4,000 human-coded tweets. The remaining 800 tweets were reserved as a testing set for evaluating all three models. The fine-tuning process aimed to enhance the model’s performance on our specific coding tasks by adjusting its parameters based on the training data. Hyperparameters for fine-tuning—including 3 epochs (training cycles), a learning rate multiplier of 1.8, and a batch size of 6—were determined by OpenAI’s API automation, reviewed and accepted after preliminary testing showed satisfactory performance. To protect privacy, only the text content of the tweets was provided to the API.

### 3.3 Performance metrics

**Figure 2:**
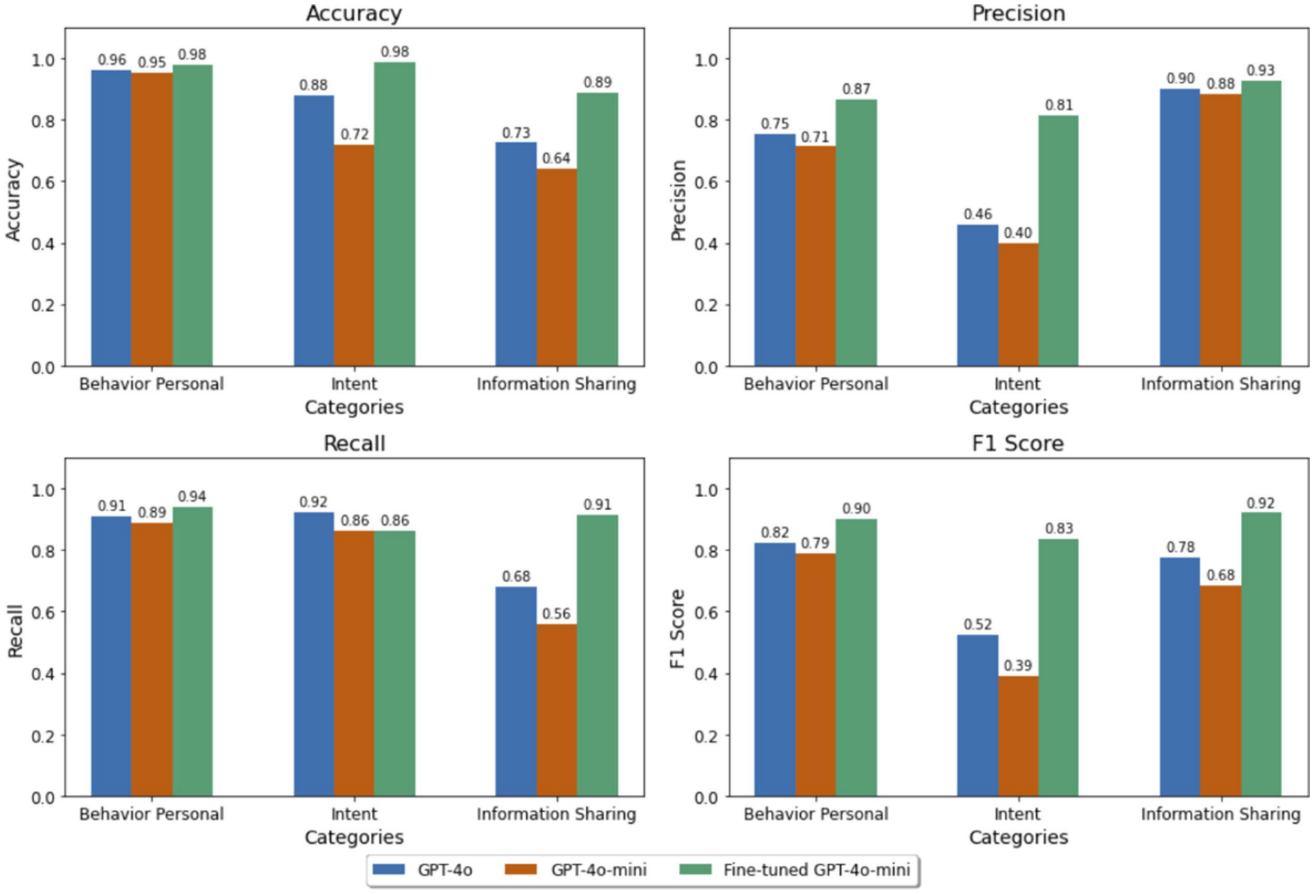
Performance metrics (accuracy, precision, recall and F1 score) across models GPT-4o, GPT-4o-mini, and GPT-4o-mini with fine-tuning.

The performance of the models was evaluated using four metrics: accuracy, precision, recall, and F1 score across three categories: behavior personal, intent, and information sharing, as depicted in Figure 2. The intent category, being a multiclass classification problem (positive, negative, or none), was assessed using the macro average method, which calculates metrics independently for each class before averaging them equally. Despite being smaller in size, the fine-tuned GPT-4o-mini model outperformed the larger GPT-4o model in most tasks. It achieved the highest scores in eleven out of twelve evaluation points (four metrics across three categories), demonstrating superior classification ability in this scenario.

As shown in Figure 2, in the behavior personal category, the fine-tuned GPT-4o-mini exhibited superior classification ability, with the highest accuracy, precision, and F1 score among all models. Similarly, in the intent category, it outperformed most models, particularly in terms of accuracy and F1 score, showcasing better precision-recall balance. Notably, while GPT-4o had slightly higher recall for Intent, the fine-tuned model’s F1 score remained substantially higher. For information sharing, the fine-tuned model demonstrated substantial improvements in all metrics, particularly in recall and F1 score, further highlighting the effectiveness of fine-tuning in improving classification performance. These results emphasize that fine-tuning GPT-4o-mini leads to performance enhancements across key content categories. This demonstrates the model’s improved capacity to accurately identify relevant instances while maintaining a balance between precision and recall.

### 3.4 Full dataset coding

Once the fine-tuned GPT-4o-mini model was identified as the best performing model based on multiple metrics (accuracy, precision, recall, and F1 score), it was selected for coding the full dataset of posts. Using this model, the remaining 371,000 tweets (full dataset excluding the 4,000 human-coded tweets) were sent to OpenAI’s API for machine coding. The same prompt used during fine-tuning was employed, ensuring consistency for the entire dataset. A custom Python script was developed to batch the data into segments of 10,000 tweets each, allowing for monitoring and addressing potential issues such as disconnection, incomplete tasks, or unexpected outputs. Unexpected JSON outputs were manually investigated, and parsing logic was implemented to successfully capture any model outputs that deviated from the expected formatting.

### 3.5 Statistical analysis

We conducted descriptive statistics on the distribution of personal behavior, intention, and information sharing, using the classification results from the fine-tuned GPT-4o-mini model. To further analyze these behaviors, a Pearson’s correlation was performed to examine the relationship between monthly tweet behaviors (e.g., sharing personal behavior, information sharing, negative intent, and positive intent) and the number of COVID-19 doses administered per month (Mathieu et al., 2021). We compared these trends over time using both correlational analysis and visual representations to validate the model’s results.

## 4 Results

The fine-tuned GPT-4o-mini model was applied to classify the full dataset across three categories: Behavior Personal, Intent, and Information Sharing. Table 1 presents the results of both human-coded and GPT-coded data, including the raw count and percentage of posts within each category, alongside example posts illustrating each content category. The behavior personal, intent (positive), intent (negative), and information sharing categories are represented with real tweet examples, coding explanations, and corresponding counts and percentages from both human coders and the fine-tuned GPT model.

**Table 1:** Raw count and frequency of human and GPT-annotated behavioral coding results with example posts across content categories.

Category	Example post	Coding explanation	Human-coded (*N*)	Human-coded (%)	GPT-coded (*N*)	GPT-coded (%)
Behavior personal	Every time I go out without a mask, as I’m fully vaccinated, I get so anxious that people are gonna think I’m an anti-masker!	The individual is sharing their personal experience of getting vaccinated, coded as “Behavior Personal.”	404	10.1%	36,912	9.84%
Intent (positive)	I just want the COVID vaccine at this point so I can move on with my life.	Expresses a positive intention to receive the vaccine, coded as “Positive Intent.”	84	2.10%	7,749	2.07%
Intent (negative)	I’m skipping this vaccine.	The user expresses a clear negative intention against getting vaccinated, coded as “Negative Intent.”	39	0.98%	3,715	0.99%
Information sharing	I’ve read that no vaccine is 100% effective, but also that the vaccine will not prevent a person from catching COVID, but will lessen possible symptoms.	The user is sharing information about vaccine efficacy, coded as “Information Sharing.”	2,789	69.73%	267,930	71.45%

### 4.1 Classification results

The fine-tuned GPT-4o-mini model identified that 9.84% of tweets (*N* = 36,912) referenced personal vaccine-related behavior, such as individuals discussing their vaccination experience or successfully obtaining a vaccination appointment, indicating that a notable proportion of users explicitly mentioned either receiving or scheduling a COVID-19 vaccine. The majority of tweets, 90.15% (*N* = 338,063), contained no explicit mention of such behaviors. These findings reflect a significant portion of users sharing personal experiences related to COVID-19 vaccinations during the studied period. Regarding vaccination intentions, 2.07% of posts (*N* = 7,749) expressed positive intentions to receive the vaccine, while 0.99% (*N* = 3,715) expressed negative intentions. The majority, 96.94% (*N* = 363,535), did not indicate any clear intent. This aligns with broader trends showing that while people engage in discussion about vaccines, few may directly declare their intent to vaccinate or refuse publicly (Dubé et al., 2014). The model detected a high prevalence of information-sharing activities, with 71.45% (*N* = 267,930) of posts disseminating information about COVID-19 vaccines. This included posts about vaccine efficacy, side effects, and logistical information about vaccination centers. The remaining 28.55% (*N* = 107,070) did not contain information-sharing content.

### 4.2 Validation of results with national COVID-19 vaccination data

**Figure 3:**
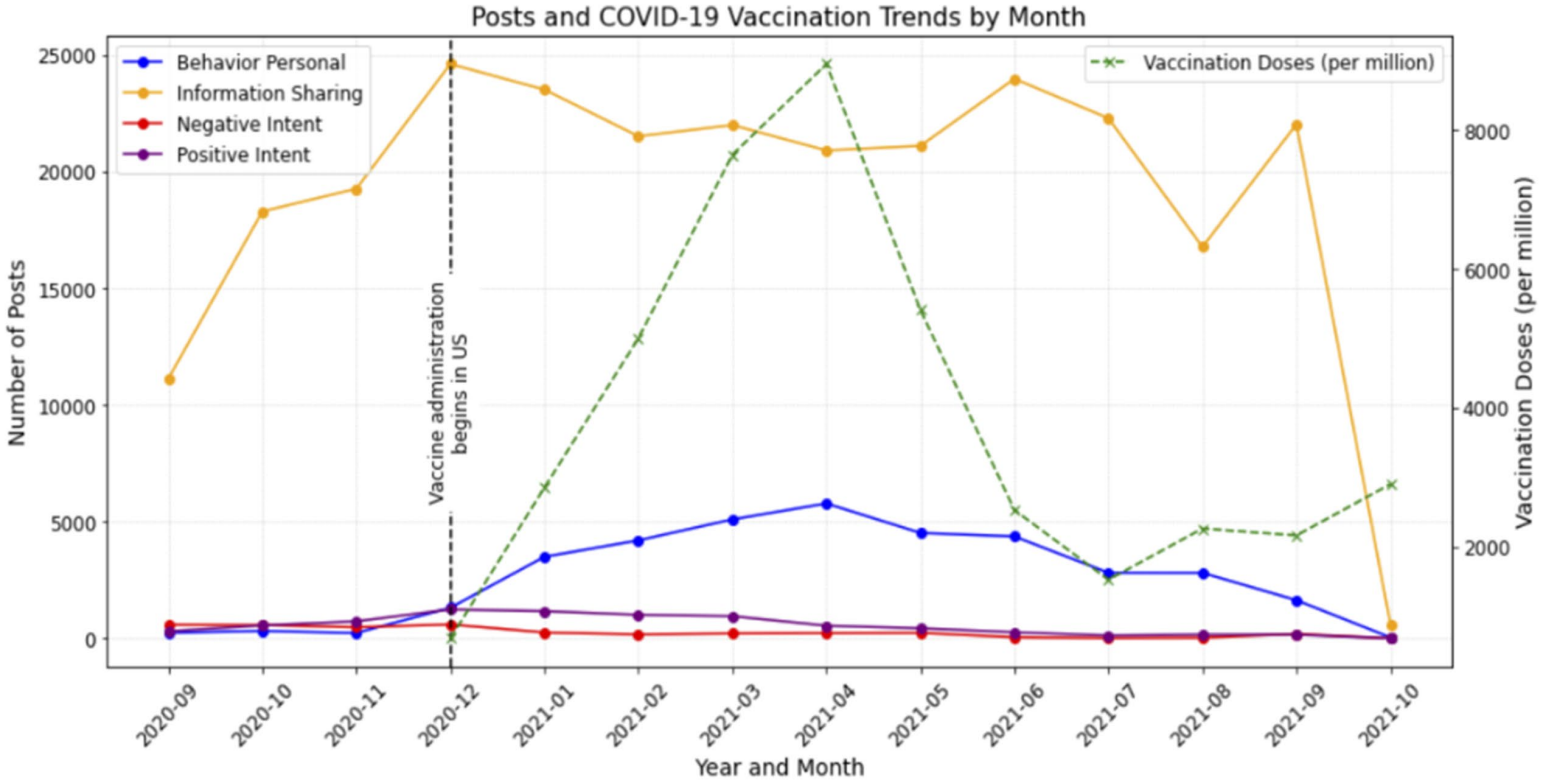
Monthly trends of coded behavioral measures (personal behavior, information sharing, and positive and negative intent) in posts and actual COVID-19 vaccination rates (doses administered per million).

To validate the model’s performance, we compared the temporal trends in vaccine-related behaviors, intentions, and information sharing identified by the model with national COVID-19 vaccination dose data (Mathieu et al., 2021). Figure 3 depicts monthly trends in social media posts related to personal behaviors, information sharing, and positive and negative intentions toward COVID-19 vaccines, alongside the average monthly COVID-19 vaccination doses administered per million people in the United States. Data on social media posts are categorized into four types: Behavior Personal, Information Sharing, and Negative and Positive Intent to get the COVID-19 vaccine. The vertical dashed line marks December 14, 2020, when COVID-19 vaccine administration began in the US. The left Y-axis represents the number of social media posts, while the right Y-axis shows the average monthly vaccine doses administered per million people.

Our findings revealed a strong correlation (*r* = 0.76, *p* < 0.01) between vaccination behaviors expressed on social media and actual vaccine uptake over time. Figure 3 depicts the number of posts per month for personal behavior, information sharing, and positive and negative intentions along with the COVID-19 vaccination rates. As shown in Figure 3, vaccination rates and online expressed vaccination behaviors displayed similar temporal patterns. Both peaked in April 2021 and declined thereafter, suggesting that LLM-based classifications can reliably capture trends in real-world vaccination behaviors. However, intent or information sharing were not correlated with COVID-19 vaccination doses over time. This suggests that while users expressed intentions regarding vaccination on social media, these sentiments may not directly correlate with real-world vaccination behaviors.

A significant positive correlation was observed between intent (negative) and intent (positive) (*r* = 0.77, *p* < 0.01), indicating that increases in posts expressing reluctance or refusal to vaccinate were correlated with increases in posts expressing positive vaccination intentions. Although personal behavior was moderately correlated with information sharing (*r* = 0.55), this relationship was not statistically significant (*p* > 0.05). This indicates that while individuals who shared their personal experiences with vaccination were also somewhat likely to share vaccine-related information, this trend was not strong enough to be definitive.

## 5 Discussion

Research has shown that LLMs can be a promising avenue for a variety of annotation tasks, offering scalability and efficiency (Gilardi et al., 2023). Our comparison of the three models revealed that the GPT-4o-mini model with fine tuning performed the highest by F1-score, indicating a hybrid approach may be advantageous for data classification. Further, the high correlation between vaccination behaviors expressed on social media and actual COVID-19 vaccination rates suggests that LLMs can be powerful tools for estimating real-world behaviors in near real-time.

Particularly in public health contexts, this approach offers a potentially more efficient and cost-effective alternative to traditional data gathering methods, allowing health officials to quickly assess trends in individuals’ health behaviors and adapt their strategies in real-time. However, the non-significant correlation between expressed intentions and actual vaccination behaviors highlights the complex nature of human decision-making. Consistent with prior research (Webb and Sheeran, 2006), this finding suggests that while social media users may express intentions to vaccinate or not, these sentiments do not translate directly into real-world actions. This intention-behavior gap may reflect the complexity of public health actions, where factors such as access, vaccine availability, or socio-political barriers play significant roles in influencing whether expressed intentions are acted upon.

### 5.1 Methodological implications

The current study has several methodological contributions. First, we employ both fine-tuning and prompt engineering in a hybrid approach. Fine-tuned models are limited in their utility as they are unable to perform well in different contexts (Hu et al., 2024), and may reinforce biases in the training data. Likewise, prompt engineering often yields unreliable results in different contexts where minute changes in phrasing can lead to significantly different outputs (Reiss, 2023). Therefore, utilizing both fine-tuning and prompt engineering can overcome these limitations, particularly when understanding human behavior. For example, we employed TOT prompting in our analyses which allows the LLM to consider multiple reasoning and thought processes. Then, we also used fine-tuning approaches to understand specific human behaviors. Overall, this hybrid approach allowed us to have a more holistic understanding of human behavior during COVID-19 and capture the complexity of behaviors efficiently. Indeed, existing work suggests that relying on both fine-tuning and prompt engineering approaches can achieve improved outcomes (Hu et al., 2024; Chung et al., 2022). This hybrid approach may contribute to future work in explainable AI, making AI data classification decisions more transparent and understandable to its users. Second, our study demonstrates how LLMs can augment human evaluation. We successfully utilized the GPT-4o-mini model to serve as an annotator, which significantly reduced time and expenses. This supports previous findings that LLMs can match or even surpass human annotators in certain tasks (Törnberg, 2023).

### 5.2 Practical implications

By observing online behaviors unobtrusively, LLM-based approaches can reduce social desirability biases and obtain objective views on the effectiveness of interventions, programs, and policies. In addition, real-time data can be captured, such as individuals sharing COVID-19 booster statuses or difficulties in booking appointments within specific communities. This information can assist health practitioners in identifying common concerns and develop targeted communication strategies, particularly in promoting COVID-19 vaccination. Here, LLMs can assist in creating messages that encourage people with positive vaccination intentions but delayed actions, providing practical information like locations of nearby free clinics. Future research may also use these large-scale behavior classification tools in combination with other API features, including geo-tagging, which can further enhance health communication efforts through tailoring.

### 5.3 Limitations

This study has several limitations. First, social media data may capture immediate, emotionally charged reactions from vocal subsets of the population, potentially amplifying certain viewpoints (Wojcik and Hughes, 2019). Further, Twitter/X is not representative of the US population, which limits the generalizability of our findings. Future research should examine different contexts to capture different populations. Second, one limitation of relying on GPT models is that they are constantly evolving and may provide different results depending on the versions (Rathje et al., 2024). For instance, an input could result in different outputs depending on the version of the model. In a similar vein, depending on the prompting approach, outputs could vary which can limit generalizability of the findings. Another limitation of using GPT models is that they require financial cost which potentially limits accessibility for researchers, particularly those from the Global South. There are also ethical concerns associated with relying on GPT models for behavioral classification. For example, inputs to GPT models are used for further training. While our study included a validation step to see if the data captured by social media data can be generalizable, integrating diverse data types and data collection methods could further mitigate biases. In addition, the inherent biases and reproducibility issues of LLMs present some challenges. LLMs function as black boxes, making it difficult to guarantee consistent results or fully explain the patterns observed in the data (Xu et al., 2024). Therefore, future research should integrate insights from established behavioral theories to guide LLM-based analyses, offering clear and reproducible models for understanding human behavior. In addition, LLMs such as GPT models exhibit cultural biases toward responses which reflect populations from contexts that are Western, Educated, Industrialized, and Democratic (WEIRD) (Atari et al., 2023). As such, researchers should exercise caution when interpreting results from LLMs and account for biases associated with WEIRD contexts.

## 6 Conclusion

This study utilized LLMs to analyze human behaviors expressed in social media posts, focusing on COVID-19 vaccine-related content during the peak of the pandemic. Our findings demonstrate that LLMs can effectively classify behaviors shared on online platforms, with our GPT-4o-mini model performing the best using a hybrid approach that combined prompt engineering with fine-tuning on targeted data. Validation of our LLM-classified behaviors revealed a significant association with actual behaviors observed in national vaccination uptake data, which underscores the potential for LLMs to inform public health strategies.

## Data Availability

The raw data supporting the conclusions of this article will be made available by the authors, without undue reservation.
