# Supplementary material for: From digital traces to public vaccination behaviors: leveraging large language models for big data classification
Source: Front Artif Intell. 2025 Jul 23;8:1602984. doi: 10.3389/frai.2025.1602984 (PMC12325327; doi:10.3389/frai.2025.1602984)
Supplement: Supplementary file 1 [file Table_1.docx]

# Supplementary material

## Supplementary Table 1.

*Coding Scheme*

| **VAX PROJECT CODEBOOK** | | | |
| --- | --- | --- | --- |
| **Behavior_Personal:** Behavior to explicitly get the vaccine personally / or successfully schedule an appointment. | | | |
| ***CODE***  None = 0 |  | | ***EXAMPLES***    · I’m forging a vaccine card  · I’m destroying a vaccine clinic. |
| ***CODE***  Yes = 1 | ***NOTES***    · Physical Actions related to vaccines.  · Volunteering (e.g., for a vaccine trial, at a vaccination site, etc.).  · Setting up a vaccine booth.  · I’m a nurse giving vaccines.  · I’m driving a friend to get vaccinated.  · This category does not include destructive/evasive vaccine related behaviors).  · PERSONALLY telling (advising) his/her family or friends about their vaccine appointments.  · Scheduling vaccine appointments for family members/friends. | | ***EXAMPLES***    · I’m in the Pfizer trial but got the placebo.  · A woman just spent 11 mins yelling at me saying she NEEDS to have her 2nd dose so she can go on vacation, and she doesn’t want to wait. MAAM what you NEED to do is to SIT THE FCK DOWN. |
| **Intent:** Behavioral Intent to get the vaccine (or not). | | | |
| ***CODE***  None = 0 | ***NOTES***    · The post does not share information about the users’ intent to get vaccinated.  · Encouraging other people to get the vaccine should be coded as “none”. This is not a personal intent to get the vaccine. Likewise, encouraging other people NOT to get the vaccine should be coded as “none”. | | ***EXAMPLES***    · @Sharkyl without a vaccine we are incredibly vulnerable and i had to supply you with those studies, don‚Äôt believe or believe idc  · I got my vaccine today!! |
| ***CODE***  Negative = 1 | ***NOTES***    · Shares intent to NOT get vaccinated/intent to avoid getting vaccinated. Expresses desire to NOT get vaccinated.  · It must explicitly express one’s wish to NOT get vaccinated. | | ***EXAMPLES***    · No Covid vaccine. I don‚Äôt get Flu shots either.<https://t.co/wpHjaryh4G>  · I’m not getting vaccinated, no way. |
| Positive = 2 | ***NOTES***    · Shares **explicit** intent to get vaccinated. Or expresses their desire to get vaccinated.  · Expressing that one is waiting for the vaccine is positive intent.  · Asking what they need to do to get the vaccine is an expression of willingness to get vaccinated.  · Expressing that you’d rather get the vaccine then die counts as intent to get the vaccine. | | ***EXAMPLES***    · Hello and I feel that WE NEED WARP SPEED FOR A VACCINE  · I can’t wait for my vaccine!!  · I’ll be happy once I get the vaccine! |
| **Info_Sharing:** Sharing information directly ABOUT COVID-19 vaccines. | | | |
| ***CODE***  None = 0 | ***NOTES***    · No information ABOUT the vaccine is shared.  · Sharing information about COVID (unrelated to the vaccine) should be coded as none (e.g., “people aren’t wearing masks they suck”).  · If the tweet is not interpretable because portions are in a non english language then code as None. | | ***EXAMPLES***    · “I just got vaccinated!! Yayy!”  · “I am not vaccinated”  · “My family hates that I’m scheduled to get my vaccine next week”  · “I’m getting my vaccine next week” |
| ***CODE***  Yes = 1  (regardless of positive or negative). | ***NOTES***    · Sharing information ABOUT the vaccine (e.g., location to get vaccinated, vaccination rates, etc).  · Sharing unfavorable, pessimistic, adverse (e.g., side-effects of vaccines), or bad information about the vaccine/getting vaccinated.  · Sharing about the benefits, helpfulness, usefulness, or advantages of the vaccine/getting vaccinated.  · Attacking/blaming others for mandating/failing to get vaccinated.  · Shares displeasure about vaccine mandates.  · Questioning the efficiency of vaccine delivery is considered information sharing.  · Sharing personal experience about getting vaccinated (e.g., what happened in the vaccine clinic, location of the site, etc.)  · Sharing that they do not want to get sick counts as info sharing.  · Talking about experience at vaccine clinic is considered info sharing.  · Talking about friends/family/self not getting vaccines before politicians is considered info sharing because it refers to vaccine distribution.  · Politicians getting a vaccine or mentions of vaccine hoax are information sharing.  · Sharing his/her family or friends’ responses to his/her vaccination should be “yes” because it contains information directly related to someone getting a vaccine/vaccine efficacy. | | ***EXAMPLES***    · Scientists Warn Pfizer, Moderna Vaccines May Cause Blood Clots, Too<https://t.co/UJtHSrZ3Tw>  · Administering an coronavirus vaccine without rigid testing and due diligence.  · Do the lives of frontline workers matter?  · #DontTrustPeopleWho recommend you to get the #vaccine  · Vaccines cause boys to act violent  · “Pfizer CEO says drug company will know if COVID-19 vaccine works before the end of October”  · “Vaccinated people can get it and spread it - FACT. The COVID vaccine is a symptom reducer - FACT.”  · @sportycanuck @ThomasStDenis3 @yycmichaelh @mat_schmaltz @PierrePoilievre How long do you want to give provinces to give shots? Qu√©bec, for example, said they could easily vaccinate 250 000 per week, so what is the problem? You think vaccines should sit in fridges for 2 months? Provinces couldn‚Äôt get their shit together?  · Vaccines and myocarditis: what do I need to know? #Pfizer #Moderna #COVID19Vaccine #myocarditis #COVIDVaccines‚Ä¶<https://t.co/nuO8fRyQiS>  · The fact that some of you won‚Äôt get the covid vaccine is so beyond selfish and privileged. You are a part of the problem. You‚Äôre why this won‚Äôt go away. The vaccine isn‚Äôt controlled by the government you morons.  · @MonicaGandhi9 Some cities line SF may have high Vax rate but others Do Not  · @LoyalistIntrov @albertmohler @BaptistPress ANY foreign missionary going overseas is required to get vaccines specific to the area of the world they are going to. Likewise, IF parents do not want their kids to get vaccinated, they have to attend private schools or homeschool. Same goes for public universities &amp; has for yrs  · @CNN And this is why I still trust J and J.  · Clots form w pfizer and Moderna too.  · 18-44 #URBAN #Bengaluru #CovidVaccine Availability for 21/08 at 07:00AM  · FREE Slots 0  · PAID Slots  · Truth. And they tweet or post on Facebook about the conspiracy theory around a tracking  · “Georgia is getting vaccines before California”  · chip being in the vaccine while holding their phones.<https://t.co/FN5k64ArXd>  · It‚Äôs Friday and my mom just got her second Covid vaccine! It‚Äôs a good day!  · 2021 will be filled with selfies of people getting the COVID vaccine, bank on it!  · My friend got vaccinated!  · My friend is not vaccinated.  · My mom got vaccinated!  · Trump got vaccinated! |
| **OTHER NOTES** | | | |
| ***COMMON COVID-19 VACCINES***  · Pfizer-BioNTech  · Moderna  · Johnson & Johnson  · AstraZeneca  · Oxford Vaccine  · Sanofi  · GlaxoSmithKline  · Novavax  · Sanofi  · Vaxart  · Symvivo  · GSK  · Covax | | ***FURTHER CLARIFICATIONS***    · I am going to get the vaccine – personal behavior.  · I got the vaccine last week – personal behavior.  · I am not going to get the vaccine – intent.  · I am waiting to get the vaccine – intent.  · Trump is behind the vaccine roll out. -- (info sharing).  · Do not trust Trump for vaccine information --(info sharing).  · Being positive/optimistic about the vaccine does not count.  · Updates about getting the shot is behavioral personal.  · Sharing others’ behavior/intention/status/responses to vaccine are info sharing. | |
|  |  |  |  |

## Supplementary Table 2.

*GPT Prompt*

| # Codebook prompt  codebook_prompt = """  You are an AI tasked with coding social media posts according to a detailed behavior codebook. Your task is to classify each post in three categories: Behavior_Personal, Intent, and Info_Sharing. For each category, output only a single digit (0, 1, or 2) based on the specific guidelines provided. Do not include any additional text or explanations.    Output the results in the following JSON format with no additional text, strictly following this format:  {  "Behavior_Personal": 0,  "Intent": 0,  "Info_Sharing": 0  }  Ensure:  - All keys are exactly as shown with no extra or missing characters.  - All values are integers (0, 1, or 2).  - No additional text, punctuation, or comments.    Follow the codebook provided below.    CODEBOOK:    1. Behavior_Personal  - Definition: Behavior to explicitly get the vaccine personally or successfully schedule an appointment.  - Coding Options:  - No = 0  - Yes = 1  - Details:  - Code 1 if the post explicitly mentions the person getting a COVID-19 vaccine or successfully scheduling an appointment to get vaccinated. Strong implications of having received dose 1 or dose 2 should also be coded as 1.  - Code 0 if the post does not explicitly mention the person got a vaccine or secured a vaccine appointment.  - Examples:  - 1: "I got the vaccine," "I scheduled my vaccine appointment."  - 0: "I got my flu vaccine today," "I tried to get an appointment but didn't get one."  - Important Notes:  - Scheduling a flu vaccine does not count as COVID-19 vaccine behavior.  - Mentioning someone else getting a vaccine is not personal behavior but information sharing.  - If the post doesn't mention which vaccine, assume it’s a COVID-19 vaccine.  - Trying to get a vaccine appointment but being unable to get it does not count as 1. Code this as 0. They have to get a vaccine or schedule an appointment to code as a 1.  - Only output a single digit, 0 or 1.    2. Intent  - Definition: Behavioral intent to get the vaccine (or not).  - Coding Options:  - None = 0  - Negative = 1  - Positive = 2  - Details:  - Code 2 if the post expresses a clear personal intent to get vaccinated or a desire to do so, including expressions of waiting for the vaccine or asking how to obtain it.  - Code 1 if the post expresses a clear personal intent not to get vaccinated or a desire to avoid vaccination.  - Code 0 if the post does not share personal intent regarding vaccination.  - Examples:  - 2: "I can’t wait to get my vaccine," "I’ll be happy once I get vaccinated."  - 1: "I’m not getting vaccinated," "No way I’m taking that vaccine."  - 0: "Everyone should get vaccinated," "You should not trust the vaccine."  - Important Notes:  - Encouraging or discouraging others from getting vaccinated should be coded as 0, as they are not explicitly personal intents.  - Only output a single digit, 0, 1, or 2.    3. Info_Sharing  - Definition: Sharing information directly about COVID-19 vaccines.  - Coding Options:  - No = 0  - Yes = 1  - Details:  - Code 1 if the post shares information directly about COVID-19 vaccines, such as vaccine locations, vaccination rates, efficacy, side effects, benefits, mandates, or personal experiences with vaccination.  - Code 0 if the post does not share information directly about COVID-19 vaccines.  - Examples:  - 1: "Pfizer vaccine causes side effects," "Vaccine slots available at my local clinic."  - 0: "My family hates that I’m scheduled to get my vaccine next week," "I am not vaccinated," "I’m getting my vaccine next week."  - Important Notes:  - General statements about COVID-19 that do not mention vaccines should be coded as 0.  - Only output a single digit, 0 or 1.    Remember:  - Output only a single digit (0, 1, or 2) for each category. Do not leave any category blank.  - Do not provide any explanations or additional text.  - The categories are mutually exclusive; select the most appropriate code based on the guidelines.    Here is the post:  """ |
| --- |

##

## Supplementary Table 3

## *Misclassification Analysis*

| **Classified Variables** | **Error Analysis** |
| --- | --- |
| Personal behavior | The non-fine-tuned models exhibited substantially more personal behavior misclassifications compared to the fine-tuned models in identifying personal vaccination behaviors. Posts containing informal language, slang, or indirect references to vaccination status were frequently misclassified by the non-fine-tuned models. For example, the post, “*kissed a girl and she had coronavirus but you know what I’m vaccinated idgaf*” was misclassified, potentially due to its casual indirect reference to vaccination status.  Fine-tuning showed remarkable improvement in handling structured data formats, successfully identifying timestamped vaccination records that the non-fine-tuned model completely missed, e.g., “*[05:37:32] 400051, 16-07-2021, 54 dose2 at BKC COVID CENTER.*”  Persistent challenges across models included metaphorical language, e.g., “*Installed ½ of the Moderna Chip*,” and posts describing others’ vaccination experiences rather than the author’s own experience. |
| Intention | Intent classification revealed a fundamental confusion between completed actions and future intentions. All models frequently misclassified celebratory posts about completed vaccinations, e.g., “*I got vaccinated today! WOOT!*” as positive intent rather than recognizing them as descriptions of completed behavior with no remaining intent.  The non-fine-tuned model struggled significantly with sarcasm and hesitancy expressed in seemingly positive language. Posts such as, “*Can’t wait to take this vaccine that was rushed through and has no long term studies,*” were misread as positive intent rather than skepticism.  Fine-tuning showed substantial improvement in detecting such sarcasm, greatly reducing misclassifications. |
| Information sharing | The non-fine-tuned model struggled in comparison to the fine-tuned model in classifying information sharing posts. The primary difficulty lay in distinguishing between personal experiences that incidentally contain information and deliberate information sharing. Clear information sharing content was frequently missed by the non-fine-tuned model, such as, “*The data of the Ministry of Health of #Bahrain confirms 94.3% efficacy and high safety*.”  All models produced some false positives by misclassifying personal celebration posts that mentioned vaccine brands, e.g., “*Thank you @DollyParton #seconddose #canadavaccinated #moderna*,” as information sharing rather than personal expression. |
